# Defect rocksalt structures in the La-Na-N system

**DOI:** 10.1098/rsta.2022.0329

**Published:** 2023-10-16

**Authors:** Yao Yuan, Simon D. Kloß, J. Paul Attfield

**Affiliations:** ^1^ Centre for Science at Extreme Conditions and School of Chemistry, University of Edinburgh, Peter Guthrie Tait Road, Edinburgh EH9 3FD, UK; ^2^ Department of Chemistry, Ludwig-Maximilians-Universität München, Butenandtstraße 5-13, Munich 81377, Germany

**Keywords:** nitrides, high-pressure chemistry, structure elucidation

## Abstract

Sodium azide (NaN_3_) is a versatile nitrogen source that can be used for the synthesis of new nitrides under high-pressure and temperature conditions. Reactions between lanthanum nitride (LaN) and sodium azide (NaN_3_) at 800°C under 8 GPa pressure have led to the discovery of two defect rocksalt phases which are the first reported ternaries in the La-Na-N system. Preliminary structure assignments have been made based on fits to powder X-ray diffraction profiles. One phase is La_1−*x*_Na_3*x*_N with vacancies at octahedral La sites and interstitial tetrahedral Na cations. This phase has a tetragonally distorted rocksalt structure (space group *I*4/*mmm*, *a* = 3.8704(2) and *c* = 5.2098(3) Å for nominal *x* = 0.10) and the distortion decreases with increasing Na content (space group *I*4/*mmm*, *a *= 3.8060(2) Å, *c *= 5.2470(3) Å for nominal *x* = 0.14), further giving a cubic phase (*a* = 5.3055(2) Å) for nominal *x* = 0.25. This coexists with another cubic $Fm\bar{3}m$ phase (*a* = 5.1561 (5) Å), tentatively identified as rocksalt ‘NaN_1/3_’ stabilized by a small amount of La; NaLa*_y_*N_(1+3*y*)/3_ with *y* ≈ 1%. These initial investigations reveal that the high-pressure La-Na-N phase diagram may be rich in defect rocksalt-type materials although further work using neutron diffraction will be needed to confirm the structures.

This article is part of the theme issue 'Exploring the length scales, timescales and chemistry of challenging materials (Part 1)'.

## 1. Introduction

Nitride materials have important applications in ceramics, optoelectronics and phosphors [1–6]. However, synthesis of solid metal nitrides is challenging because of the stability and inertness of the N_2_ molecule. High-pressure high-temperature (HPHT) reactions can be used to overcome this issue as illustrated by the notable work of the late Paul McMillan in preparing new high-pressure metal nitrides [7–10]. A recent development has been the use of sodium azide (NaN_3_) as a versatile nitrogen source in HPHT reactions that can lead to the synthesis of stoichiometric ternary nitrides of transition metals in high-oxidation states such as Ca_4_FeN_4_ [11], Ca_2_NiN_2_ [12] and the perovskite LaReN_3_ [13,14]. Decomposition of NaN_3_ results both in a high nitrogen activity and metallic Na to act as a flux, but there is the possibility that Na could be incorporated into a product phase. No Na was found within the latter materials or other reported products of HPHT reactions with NaN_3_ such as rocksalt-type Mg_0.4_Fe_0.6_N [15], but we have investigated this possibility through HPHT reactions of NaN_3_ with the simple nitride LaN to discover whether ternary Na-containing products can be formed.

LaN adopts the rocksalt-type structure in space group $Fm\bar{3}m$ (No. 225), although a tetragonal variant was reported from a high-pressure study [16]. Rocksalt-type nitrides like LaN have historically been investigated as ceramic abrasives and coatings as well as superconductors but have also recently gained attention as semiconductors [17]. Many ternary nitrides have been derived from LaN, for example, ‘La_6_Cr_21_N_23_’, which shows superconductivity below 2.7 K [18], and was later shown to be rocksalt-related La_3_Cr_10−x_N_11_ [19], the La_3_M_2_N_6_ (M = Cr, V, Nb, Ta) series [20–22] and La_2_GaN_3_ [23]. The present study shows that two distinct non-stoichiometric defect rocksalt phases, the first reported La-Na-N ternaries, can be formed through HPHT reactions.

## 2. Experimental section

### (a) Sample preparation

All reagents and products are very sensitive to air and moisture and so were handled in an argon-filled glove box with no direct air exposure. Mixtures of LaN powder (LaN, Alfa Aesar 99.9%) and sodium azide (NaN_3_, Aldrich 99.5%) were sealed in a copper container. All samples were treated under HPHT conditions using a Walker-type multi-anvil apparatus. Samples were compressed to 8 GPa pressure, heated at 800°C for 3 h and cooled to room temperature in 2 h, followed by slow pressure release to ambient. The LaN : NaN_3_ molar ratios investigated were 1 : 0, 1 : 1/3, 1 : 1/2, 1 : 1 and 1 : 2, corresponding to *x *= 0, 0.10, 1/7(≈0.14), 0.25 and 0.4 in ideally stoichiometric La_1−*x*_Na_3*x*_N compounds.

### (b) Powder diffraction

Powder X-ray diffraction (XRD) was carried out using a Bruker D8 Advance powder diffractometer with monochromated Cu-*K_α_*_1_ radiation (wavelength *λ* = 1.5406 Å). Samples were loaded into borosilicate glass capillaries (*ϕ* = 0.5 mm) filled with dry argon gas. Data were collected over angular range 2*θ = *5–90° in steps of 0.01°. Rietveld profile fits were performed with the FullProf software package [24]. *B*_iso_ thermal parameters for all atoms were constrained to the same values (or fixed in some cases) to lessen correlations with absorption and site occupation factors. However, wide variations in *B*_iso_ values are still observed due to such correlations. Anisotropic strain or stacking fault broadening of some diffraction peaks is also observed in the Rietveld fits, but this was not analysed. Although the XRD is dominated by La and so is relatively insensitive to N and Na atoms, the refinements can indicate which of several possible defect models is more likely through comparison of fits using the same thermal, lattice and profile parameters.

## 3. Results

### (a) LaN-NaN_3_ reaction products

**Figure 1.**
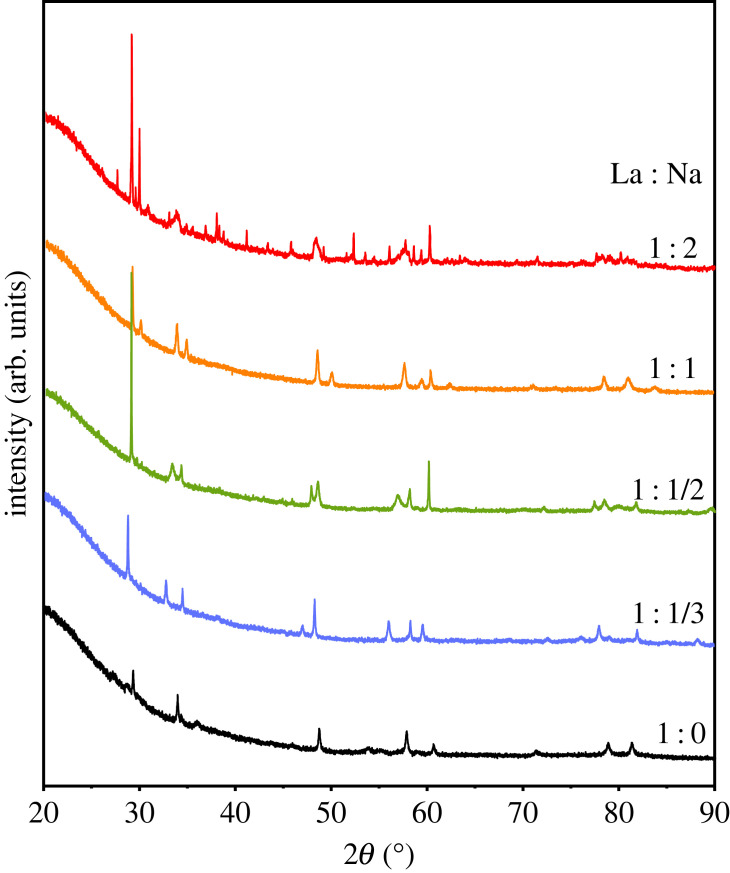
XRD patterns of products from HPHT reaction of LaN and NaN_3_ in the ratios shown. (Online version in colour.)

Powder XRD patterns of HPHT products from reaction of LaN and NaN_3_ in varying proportions are shown in figure 1. All products were dark grey powders that were sensitive to moisture and air. HPHT treatment of LaN without any sodium azide was also carried out as a check. A Rietveld fit of a stoichiometric LaN model to this XRD profile (shown in the electronic supplementary material, figure S1) gave a refined lattice parameter *a*_*c*_ = 5.2928(3) Å, close to the reported value of 5.302 Å for LaN [16,25]. This demonstrates that LaN is unaltered under the present HPHT conditions, so that changes to its XRD pattern after heating with sodium azide seen in the other patterns in figure 1 demonstrate that La-Na-N reaction products are present. The LaN-2NaN_3_ sample was found to contain a complex mixture of phases and was not analysed further. Simple products in two distinct regimes were observed for other samples, as described in the following sections.

### (b) Tetragonal La_1−*x*_Na_3*x*_N phases (*x* = 0.10 and 0.14)

After the HPHT reaction, LaN-1/3NaN_3_ (nominally La_1−*x*_Na_3*x*_N with *x* = 0.10) and LaN-1/2NaN_3_ (*x* = 0.14) samples were found to contain a single tetragonal phase with a rocksalt-related structure and cell parameters $a\approx a_{c}/\sqrt{2}$ and *c* ≈ *a*_*c*_ where $c<\sqrt{2}a$. This is different to the only previously reported tetragonal form of LaN where the lattice distortion has $c>\sqrt{2}a$ [15] and the X-ray patterns also do not match those of known high-pressure LaN or NaN_3_ or Na_3_N phases [26–29].

**Figure 2.**
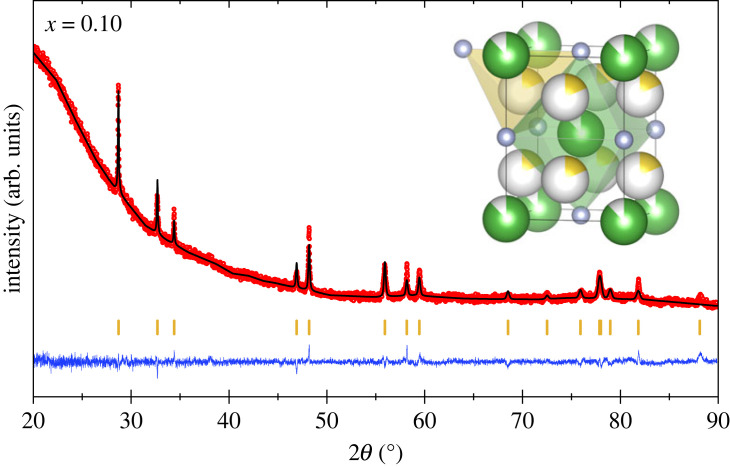
Rietveld fit to XRD data for the tetragonal °La_1−*x*_*^t^*Na_3*x*_N (*x* = 0.10) phase. The inset structure shows cation sites as large spheres (octahedral 88% occupied by La; tetrahedral 18% occupied by Na in *x* = 0.10 sample) and N as small spheres. (Online version in colour.)

Rocksalt-derived LaN-Na_3_N nitride solid solutions have an excess of cations over anions compared with LaN. This can be accommodated by having excess cations in tetrahedral interstitial sites (i.e. La_1−*x*_Na_3*x*_N, known as the LiPd_2_Tl-type or Li_3_Bi-type structures when all octahedral and tetrahedral sites are filled), or by formation of anion vacancies within a NaCl-type lattice, i.e. La_1−*y*_Na*_y_*N_1−2*y*/3_. A similar vacancy model was previously reported for defect La-Ca-N rocksalt nitrides La_1−*x*_Ca_*x*_N_1−*x*/3_ [30]. Refinement of the corresponding La_1−*y*_Na*_y_*N_1−2*y*/3_ vacancy model against the present XRD data gave a goodness-of-fit *χ*^2 ^= 2.2 for the *x* = 0.10 sample. La_1−*x*_Na_3*x*_N cation-interstitial models were also tried. Assuming that the octahedral cation site remains full with excess Na on the tetrahedral interstitial sites (i.e. °(La_1−*x*_Na*_x_*)^t^Na_2*x*_N where o/t superscripts represent octahedral/tetrahedral sites) gave *χ*^2 ^= 2.1. However, a model in which all Na ions occupy the interstitial tetrahedral sites while La-vacancies are formed at the octahedral sites (°La_1−*x*_^t^Na_3*x*_N) gave the lowest goodness-of-fit (*χ*^2 ^= 1.7) for the *x* = 0.10 XRD profile, shown as figure 2. As well as giving the best fit, the latter model is more realistic than the former as the °(La_1−*x*_Na*_x_*)^t^Na_2*x*_N structure has short, highly unfavourable, octahedral La^3+^/Na^+^ to tetrahedral Na^+^ cation distances of 2.3 Å which can be avoided in the °La_1−*x*_^t^Na_3*x*_N alternative by clustering of the Na interstitials around La vacancies. The °La_1−*x*_^t^Na_3*x*_N model also gave the best fit to the *x* = 0.14 XRD profile, shown in the electronic supplementary material, figure S2.

The °La_1−*x*_^t^Na_3*x*_N model is thus proposed as the most likely structure for the tetragonal phase. Refinement parameters for the LaN-1/3NaN_3_ (*x* = 0.10) and LaN-1/2NaN_3_ (*x* = 0.14) samples are shown in table 1. The refined values of *x* = 0.122(4) and 0.170(6) are in good agreement with the ideal compositions of *x* = 0.10 and 0.14, respectively, confirming the usefulness of the powder XRD refinements.

**Table 1.** Atomic parameters and interatomic distances for tetragonal *I*4*/mmm* °La_1-*x*_*^t^*Na_3*x*_N phases (upper values *x* = 0.10, lower values *x* = 0.14) refined against XRD data. (For *x* = 0.10; *a* = 3.8704(2) and *c* = 5.2098(3) Å; residuals *R*_p_ = 2.16%, *R*_wp_ = 3.04%, *R*_B_ = 8.49%, *R*_f_ = 4.82%, *χ*^2^ = 1.66. For *x* = 0.14; *a* = 3.8060(2) and *c* = 5.2470(3) Å; residuals *R*_p_ = 3.06%, *R*_wp_ = 4.91%, *R*_B_ = 13.3%. *R*_f_ = 8.74%, *χ*^2^ = 3.60.)

site	*X*	*y*	*z*	Occ	*B*_iso_ (Å^2^)
La (2a)	0	0	0	0.878(4)	3.62(7)
				0.830(6)	1.96(8)
Na (4d)	0	0.5	0.25	0.184	3.62
				0.253	1.96
N (2b)	0	0	0.5	1	3.62
					1.96

bond	length (Å)	bond	length (Å)		
(La-N) x4	2.7367(1)	(Na-N) x4	2.3326(1)		
		2.6913(1)	2.3113(1)		
(La-N) x2	2.6048(1)	(Na-La) x4	2.3326(1)		
		2.6235(2)	2.3113(1)		

### (c) Two-phase behaviour at *x* = 0.25

After HPHT reaction, the XRD pattern of the LaN-NaN_3_ sample (equivalent to average composition La_1−*x*_Na_3*x*_N with *x* = 0.25) shows peaks from two face-centred cubic (fcc) $Fm\bar{3}m$ phases with lattice parameters *a* = 5.3055(2) Å (Phase 1) and *a* = 5.1561(5) Å (Phase 2). The former is very close to the value for pure LaN, and so the intensities for Phase 1 were fitted using the same ^o^La_1−*x*_^t^Na_3*x*_N model as applied to the tetragonal phases above but in cubic space group $Fm\bar{3}m$. Results in table 2 reveal cation occupancies similar to those in the above tetragonal phases.

**Table 2.** Atomic parameters and interatomic distances for the cubic °La_1−*x*_^t^Na_3*x*_N Phase 1 in the nominal-*x* = 0.25 sample refined against XRD data ($Fm\bar{3}m$; *a* = 5.3055(2) Å; residuals *R*_p_ = 2.24%, *R*_wp_ = 3.08%, *R*_B_ = 8.04%, *R*_f_ = 4.34%, *χ*^2^ = 1.43).

site	*X*	*y*	*z*	Occ	*B*_iso_ (Å^2^)
La (4a)	0	0	0	0.868(4)	0.19(6)
Na (8c)	0.25	0.25	0.25	0.198	0.19
N (4b)	0.5	0.5	0.5	1	0.19
bond	length (Å)	bond	length (Å)		
(Na-N) x4	2.2973(1)	(Na-La) x4	2.2973(1)		
(La-N) x6	2.6527(1)				

The cell volume of the additional cubic Phase 2 is approximately 10% less than that of LaN and the cation-interstitial °La_1−*x*_^t^Na_3*x*_N phases above, implying that some different structure or defect mechanism involving less atoms in the unit cell pertains. Attempts to refine other simple cubic structures such as fluorite or spinel gave poorer fits than rocksalt-type models. The rocksalt-type La_1−*y*_Na*_y_*N_1−2*y*/3_ anion vacancy model gave unrealistically large refined N occupancies (greater than 100%) for La-rich cation compositions. However, with only Na at the cation site of the rocksalt lattice, the N site occupancy refined to 0.38(2), which is near to the ideal value of $\frac{1}{3}$ for a rocksalt-type form of Na_3_N with N-vacancies, i.e. NaN_1/3_. Attempts to synthesis this phase from HPHT decomposition of NaN_3_ with no LaN present were unsuccessful, with the main product being body-centred cubic Na metal as found previously [31], so it was concluded that Phase 2 is ‘NaN_1/3_’ stabilized by incorporation of a small amount of La. This was corroborated by a repeat HPHT reaction of the same LaN-NaN_3_ composition which gave a significantly different cubic cell parameter value for Phase 2 (*a*_2_ = 5.1183(3) Å compared with *a*_1_ = 5.1561(5) Å for the first run), showing that it has variable composition.

**Figure 3.**
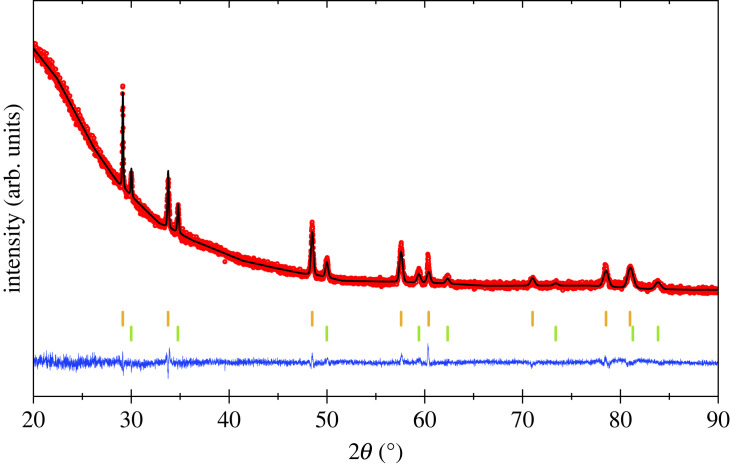
Rietveld fit to XRD data for the *x* = 0.25 sample showing cubic °La_1−*x*_^t^Na_3*x*_N Phase 1 and cubic °Na^t^La_y_N_(1 + 3*y*)/3_ Phase 2 as upper and lower markers, respectively. (Online version in colour.)

Determination of the mechanism for La incorporation in Phase 2 is not straightforward as the La content is small. Refinement of La at the Na sites, i.e. °(Na_1−*y*_La*_y_*)N_(1 + 2*y*)/3_ gave a negative La occupancy, but placing La at the interstitial tetrahedral sites according to °Na^t^La*_y_*N_(1 + 3*y*)/3_ gives a small positive La-site occupancy corresponding to *y* = 0.012(2). The latter model might be expected to have octahedral site vacancies to avoid short cation-cation contacts, as was found for the cation-excess °La_1−*x*_^t^Na_3*x*_N phases above, but it is not possible to refine occupancies at all sites as one site content has to be fixed for the structure refinement to be stable, and so °Na^t^La*_y_*N_(1+3*y*)/3_ was taken as the best description for the structure of Phase 2 available from the present XRD study. Results are shown in table 3 and the electronic supplementary material, table S3, and the overall profile fit is shown in figure 3 and the electronic supplementary material, figure S3. The apparent La-N distance of 2.23 Å is very short (cf. 2.65 Å in LaN) but this likely reflects averaging over mostly empty tetrahedral sites and the very small faction of sites (0.6%) occupied by La where local rearrangements to give longer La-N bonds may occur.

**Table 3.** Atomic parameters and interatomic distances for the cubic rocksalt °Na^t^La*_y_*N_(1+3*y*)/3_ Phase 2 in the nominal-*x* = 0.25 sample refined against XRD data, with free refinement of the La and N site occupancies ($Fm\bar{3}m$; *a* = 5.1561(5) Å; residuals *R*_p_ = 2.24%, *R*_wp_ = 3.08%, *R*_B_ = 23.8%, *R*_f_ = 13.2%, *χ*^2^ = 1.43). The refined Phase 1 : Phase 2 weight fraction ratio was 37(2) : 63.

site	*x*	*y*	*z*	Occ	*B*_iso_ (Å^2^)
Na (4a)	0	0	0	1	0.13(16)
La (8c)	0.25	0.25	0.25	0.006(1)	0.13
N (4b)	0.5	0.5	0.5	0.346	0.13
bond	length (Å)	bond	length (Å)		
(La-N) x4	2.2326(3)	(La-Na) x4	2.2326(3)		
(Na-N) x6	2.5780(3)				

The *x* = 0.25 composition is thus found to consist of a mixture of two distinct defect rocksalt phases: La-rich, cation-excess °La_1−*x*_^t^Na_3*x*_N Phase 1 with *x* < 0.2, and the Na-rich, anion-deficient °Na^t^La*_y_*N_(1 + 3*y*)/3_ Phase 2 where *y* ≈ 0.01. The difference between their La/Na ratios likely reflects a large chemical miscibility gap that gives rise to the observed two-phase behaviour.

## 4. Discussion

Rocksalt-related metal oxides have a great variety including cation-ordered ABO_2_, A_2_BO_3_, A_3_BO_4_ and A_5_BO_6_ types [32], and non-stoichiometric materials with rich defect chemistries such as wűstite, Fe_1−*x*_O. No Na/La cation-ordered phases are apparent in this HPHT investigation of the La-Na-N system, but an interesting series of non-stoichiometric rocksalt phases have been discovered. These are recovered as metastable materials under ambient conditions.

**Figure 4.**
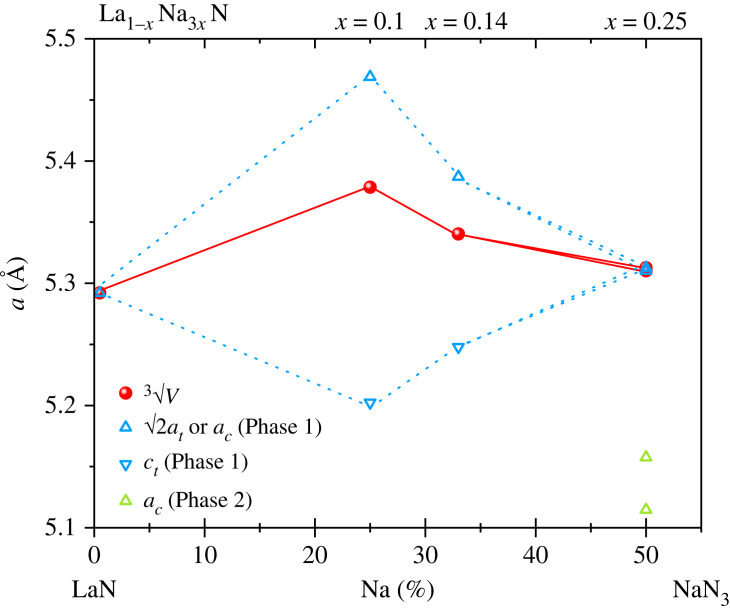
Variation of product cell parameters with composition of LaN-NaN_3_ precursors. For ease of comparison, parameters are normalized to the rocksalt cubic parameter. (Online version in colour.)

Changes of structure are summarized on the lattice parameter plot shown in figure 4. It can be seen that a small Na content (*x* = 0.10) gives the maximum observed tetragonal distortion in the cation-interstitial °La_1−*x*_^t^Na_3*x*_N phase. Investigation of further samples in the *x* = 0–0.10 range will be needed to discover the onset of the tetragonal distortion from cubic at *x* = 0. As the Na content increases, the distortion becomes smaller in *x* = 0.14 and the system regains cubic symmetry at *x* = 0.25. However, the refined *x* = 0.132(4) and *x *= 0.170(4) values are higher than would be expected from the trend of the two tetragonal materials, and this may reflect correlations in the two-phase refinement for *x* = 0.25. A second non-stoichiometric phase emerges at *x* = 0.25, this appears to be a La-stabilized form of rocksalt-type ‘NaN_1/3_′ with N-vacancies. A similar (but tetragonal) ‘CuN_1/3_’ structure was reported for a high-pressure form of Cu_3_N [33]. This structure type has not been previously reported among polymorphs of Na_3_N and HPHT decomposition products of NaN_3_ [31] and present results suggest that it can be stabilized by approximately 1% La doping as the pure binary rocksalt-type NaN_1/3_ was not obtained under the same HPHT conditions. This is not surprising as Na_3_N is known to be thermally unstable above 360 K at ambient pressure, requiring an indirect synthesis route to obtain the ordered anti-ReO_3_ type form [34]. Addition of a small amount of La (in unusual tetrahedral nitride coordination in interstitial sites) thus appears to be a useful strategy for recovery of a slightly doped rocksalt-type ‘NaN_1/3_’ structure to ambient conditions.

All of the compositions assigned in this study are based on comparison of powder X-ray refinements, which may not be accurate for light Na and N atoms in the presence of heavy La, and so further neutron diffraction studies will be useful to confirm the structures and explore further aspects such as local clustering of interstitials and vacancies. Investigation of further compositions and at other pressures and temperatures will be needed to map out what may be a rich phase diagram of defect rocksalt-type materials in the La-Na-N system. Such materials could be investigated as Na-ion conductors, although their air-sensitivity may preclude practical applications. It will be also interesting to compare La-Na-N against other nitride systems such as La_1−*x*_Ca_*x*_N_1−*x*/3_ where anion vacancy formation was reported [30], in contrast to the cation interstitials for La-rich °La_1−*x*_^t^Na_3*x*_N discovered here, and against the many studied oxide rocksalt systems.

## 5. Conclusion

In summary, two ternary defect rocksalt phases have been discovered in the La-Na-N system through HPHT reaction. These nitrides are stable at ambient pressure under inert atmospheres, but are air and moisture sensitive. La_1−*x*_Na_3*x*_N has vacancies at octahedral La sites and interstitial tetrahedral Na cations. This phase has a tetragonally distorted rocksalt structure at low *x* and the distortion decreases with increasing Na content, finally giving a cubic $Fm\bar{3}m$ phase for nominal *x* = 0.25. The latter composition coexists with another cubic rocksalt-derived phase with a smaller cell parameter, based on a rocksalt form of Na_3_N with N-vacancies, ‘NaN_1/3_’, that has been modelled as NaLa*_y_*N_(1+3*y*)/3_ with a small amount of La (*y* ≈ 1%) at interstitial sites. These investigations reveal that the high-pressure La-Na-N phase diagram may be rich in defect rocksalt-type materials.

## Data Availability

Data are in paper and electronic supplementary material [35].
